# The Effect of *Melissa Officinalis *Extract on the Severity of Primary Dysmenorrha

**Published:** 2017

**Authors:** Parvaneh Mirabi, Mahshid Namdari, SeidehHanieh Alamolhoda, Faraz Mojab

**Affiliations:** a *Infertility and Reproductive Health Research Center, Health Research Institute, BabolUniversity of Medical Sciences, Babol, Iran*; b *Department of Biostatistics, Faculty of Paramedical Sciences, ShahidBeheshti University of Medical Sciences, Tehran, Iran. *; c *Department of Reproductive Health and Midwifery, School of Nursing and Midwifery, ShahidBeheshti University of Medical Sciences, Tehran, Iran. *; d *Pharmaceutical Sciences Research Center and School of Pharmacy, ShahidBeheshti University of Medical Sciences, Tehran, Iran.*

**Keywords:** Primary dysmenorrhea, *Melissa officinalis*, Spasm, Pain, Clinical trial

## Abstract

Primary dysmenorrhea refers to painful cramps during menstruation with no organic reason. With respect to its high incidence and adverse outcomes in quality of life and some evidences regarding the sedative and antispasmodic effects of *Melissa officinails*on smooth muscles as an herb, this double-blind clinical trial was conducted to determine the effects of its capsules on severity of dysmenorrhea in the students of Islamic Azad University of Zanjan in 2014.

110 students were matched in terms of dysmenorrhea severity and experience; age; menarche; body mass index (BMI); occupation as well as educational level of parents; and duration, interval as well as amount of bleeding. Then, they were randomly divided into 2 herb (55 subjects) and placebo (55 subjects) groups. The former was given capsules 330 mg of the herb 3 times a day over 3 days at the onset of hemorrhage while the latter was given placebo in similar capsules containing corn starch with the same protocol. Pain severity was evaluated with a visual analogue scale (0 to 10 cm). Different statistical tests were used for data analysis with SPSS package.

No significant difference was found between the means of pain severity in the groups before the intervention. However, the severity was reduced in both groups after the intervention (P<0.001) but the amount of it was more in Melissa group with a significant difference (P<0.05). With respect to the findings, it seems that *M. officinalis*may decrease dysmenorrhea, which may be related to antispasmodic effects of this herb.

## Introduction

Primary dysmenorrhea, a common gynecological disorder classified as painful cramps during menstruation that occurs in the absence of identifiable pelvic disorder (1).

It is mostly confined to the adolescent age group, appearing after the menarche and less often in women after the age of 20-25 years (2).

Dysmenorrhea is a common cause of absenteeism from work and school andhas a negative impact on the quality of life and general health of women(3).

Menstrual pain resulting from increased prostaglandins (PGs), that stimulates uterine contractions, decrease uterine blood flow, and increase peripheral nerve hypersensitivity, increases vasopressin and leukotriene release, which leads to ischemia and pain(4).

The prevalence of dysmenorrhea is high among female adolescents (50-70%) (2), in Iran, its incidence has been reported between 71 and 89.9 % (5, 6).

Non-steroidal anti-inflammatory drugs (NSAIDs) such as Mefenamic acid, Ibuprofen, ketoprofen, naproxen, and celecoxibare commonly used in women with dysmenorrhea (7).

Other treatment options include oral contraceptives to prevent ovulation or hormonal drugstranscutaneous, electrical nerve stimulation, Acupressure (8)and acupuncture,β-blockers, presacralneurotomy and hysterectomy, etc (9).World-wide use of herbal medicines is increasing, following regulatory and manufacturing developments. Herbs are attractive alternative medications to many patients with dysmenorrhea who may be averse to using conventional drugs.Therefore, many traditional lyused herbs have been investigate dinmany different *in-vitro* and *in-vivo* studies.

*Melissa officinalis*L. (Lemon balm), a valuable medicinal plant in herbal medicine is native to the eastern Mediterranean Region and western Asia(10). The constituent of the essential oil of the plant in various climates is different, but citral, citronellal,and geraniol are main components. Lemon balm has been traditionally used for different medical purposes as tonic, antispasmodic, carminative, diaphoretic, surgical dressing for wounds, sedative-hypnotic, strengthening the memory, and relief of stress induced headache, but in modern pharmacology is value in the management of mild to moderate Alzheimer's, against migraine, rheumatism, and antioxidant activities (11).

*M. officinalis* (MO) has been used in aromatherapy, mostly because of their analgesic, anxiolytic, spasmolytic effects on headaches, migraines, rheumatic pains, mood, sexual, immune disorders and menopause(12, 13).

The antioxidant activity of MO was evaluated to understand the mechanism of its pharmacological properties as well as its potential genotoxic and cytotoxic effects on human leukocytes.

The results showed strong reducing power and exhibited a significant inhibition of deoxyribose degradation. MO interfered with the formation of 1,10-phenanthroline-Fe^2+^ complex, suggesting that it has chelating activity and captures Fe^2+^ before 1,10-phenanthroline. MO was neither genotoxic nor cytotoxic at the concentrations tested, indicating that the popular use of the extract might possibly not result in any genotoxic or cytotoxic effects. Results suggest that MO is a potential source of natural antioxidants, and could be relevant to the management of oxidative stress(11). In some articles, *M. officinalis* is cited as useful hypnotic (14, 15).

The essential oil obtained from leaves of *M. officinalis* was investigated for its chemical composition and *in-vitro* antimicrobial activity. The major component was geranial (44.2%). Other predominant components were neral (30.2%) and citronellal (6.3%). The *in-vitro* antimicrobial activity was determined by paper disk agar diffusion testing and minimum inhibitory concentration (MIC) using 7 bacteria, 2 yeasts and 3 fungi. The results showed that the essential oil presented high antimicrobial activity against all microorganisms targeted (16). Relaxant effects of lemon balm oil were investigated on tracheal and ilea smooth muscles of the guinea pig and it was the most potent (17).

According to above properties, it seems that lemon balm can prevent or reduce dysmenorrhea pain. Since no scientific evidence was found in this connection, this double- blind clinical trial was conducted to examine possible antispasmodic effect of the *Melissa officinalis* extract on the severity of primary dysmenorrhea of the students in Islamic Azad University students in Zanjan (Province of Zanjan, Iran).

## Experimental

This project was approved 24 of December 2011 by ethical committee of Shahid Beheshty University of Medical Sciences with No. 89-01-86-7505-6498, and it was registered in Iranian Registry of Clinical Trial (IRCT201203045975N3).

Sample size formula and expected power are as:

$1-\beta =0.80\Rightarrow z_{\beta }=0.84$

$n=2\frac{{\left(Z_{\alpha }+Z_{\beta }\right)}^{2}\sigma^{2}}{{\left(\mu_{1}-\mu_{2}\right)}^{2}}$

All single students at dormitory with dysmenorrhea participated in the study. Sample size was estimated 110 subjects with respect to similar studies with 95% confidence interval. The purpose and methods of the study were first described for the students and a written informed consent was then taken. A questionnaire in 2 parts was used for data collection.

The first part was completed before the intervention and the second part including 2 data forms regarding the peak of pain severity during menstrual cycle and its associated systemic signs was completed by the students 2 times over 2 consecutive cycles. Content and test-retest methods were used for validity and reliability (r=0.9) of the questionnaire respectively.

 Demographic data included age, body mass index (BMI), educational level, occupation of parents, exercise program, stressful factors in the past 6 months, number of sedative drugs taken during dysmenorrhea and a pain severity chart.

The severity of dysmenorrhea was measured by a visual analogue scale (a 10-cm band) denoting 1-3 as weak, 4-7 as moderate and 8-10 as severe pain (18). In addition, a multidimensional verbal scoring system from 0 to 3 was used for associated systemic signs (19).

Inclusion criteria included: single students (18-25 years old) at dormitory with primary dysmenorrhea.History of specific diseases, consumption of drugs, burning, itching, discharge, stressful factors in the past 6 months, irregular menstrual cycles and mild dysmenorrhea (scores 1-3) were regarded as exclusive criteria. The eligible subjects were equally allotted to separate blocks (moderate and severe dysmenorrhea) to complete each with 50 students.

Because of attrition, 55subjects were selected for each group to make 110students participate in the study. The subjects were matched in terms of dysmenorrhea severity and then randomly (by software) divided into 2 experimental and placebo groups.

MO samples were prepared from cultivated farms around Karaj(Province Alborz) in December 2013 and, after identification and verification in School of Pharmacy, Shahid Beheshti University of Medical Sciences, Tehran, grinded by electrical grinder. The resultant powder was extracted with ethanol 96% ( 3), the extract mixed with corn starch and then powdered extract was put into capsules (size 000) with handy machine. The placebo capsules containing corn starch were made through the same conditions. For the Melissa group, capsules containing 330 mg extract of herb were given for 3 days from the beginning of menstruation 3 times daily over 2 cycles. The placebo group was given capsules containing corn starch with the same protocol. The capsules were similar in shape and package.

Data regarding pain severity, menstruation and sedative consumption were collected by forms to be completed in 2 cycles. Pain severity was recorded 3 times a day (the most severe pain) in the forms. In case of sedative consumption, the subjects were asked to take the drug 1 hour after having Melissa capsules and to document pain severity before the consumption. The capsules were administered with codes and findings were documented in a separate form. Both subjects and researchers were uninformed of the groups. SPSS package (ver. 16) was used for data analysis. Descriptive data were presented in different tables of frequencies, means and standard deviations to demonstrate the demographic characteristics of the subjects. Friedman statistical test was first used for comparing pain severity between 3 cycles and Mann Whitney test for comparing findings between the 2 groups. In significant results of Friedman test, therapeutic cycles were compared in pairs by modification of α and Wilcoxon test.

## Results

**Figure1:**
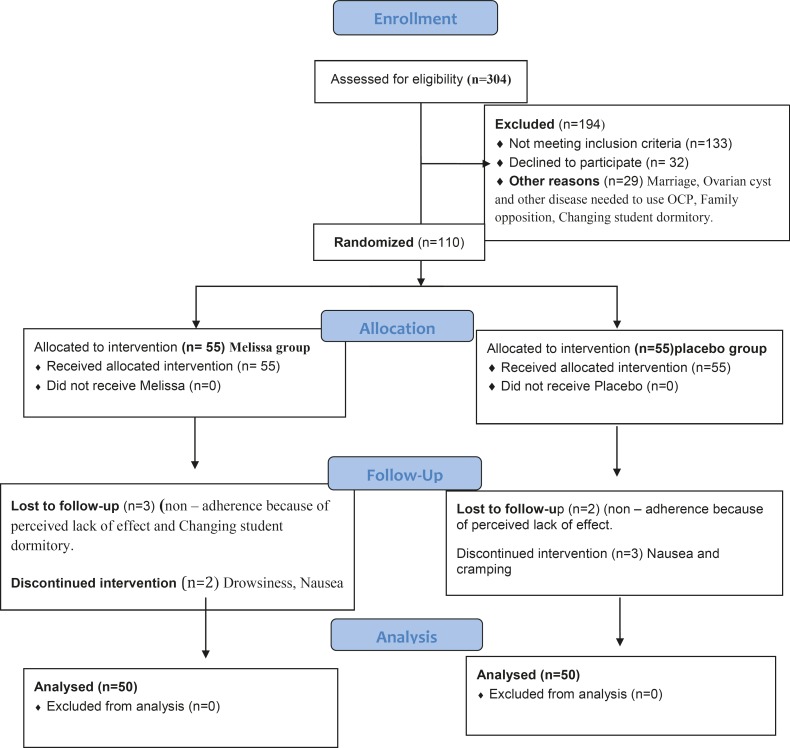
Flow of participants through the study.

Of 620 single female dormitory residents, 304 reported primary dysmenorrhea (Figure 1, consort). After exclusions, 110 individuals were enrolled in the study. The final analysis included 100 women, 50 of whom received Melissa and 50 received placebo. 

There were no significant differences between the groups in terms of age, age at menarche, age at onset of dysmenorrhea, and body mass index (Table 1).

The pain severity at baseline did not differ significantly between the groups (p=0.267).

**Figure 2:**
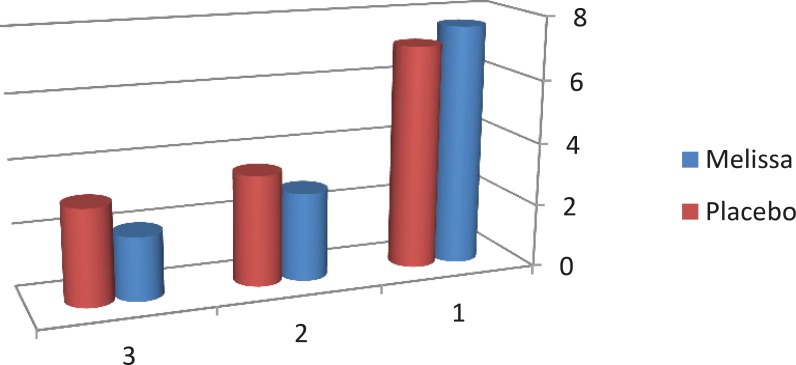
Mean changes in severity of dysmenorrhea in Melissaofficinalis and placebo groups before and after the second cycle

According to the results, the pain severity in the Melissa group decreased from 7.63 to 2.00 and in the placebo group from 7.09 to 3.03 in the second cycle. In addition, a significant difference between the 2 groups was found in the first and second cycle in terms of pain severity (p<0.001) (Table 2, Figure 2).

The pain severity in each intervention cycle differed significantly between the 2 groups, with pain reduction in each cycle being significantly larger in the Melissa group (Table 3). 

The mean numbers of sedative tablets in the Melissa group decreased from3.25 before the intervention to 2.12 in the first and to 1.20 in the second cycle.

The corresponding figures in the placebo group were 3.23, 2.59, and 2.38, respectively. The decrease was significantly larger in the Melissa group (p=0.001).

**Table 1:** Demographic characteristics of the participants

**Characteristics**	**Melissa**	**Placebo**	**P value**
Age^a^(year)	21.08±1.34	21.14±1.61	0.90
Menarche	13.30±1.35	13.46±1.05	0.55
Age of dysmenorrhea^a^	15.62±2.23	15.66±1.84	0.79
Body mass index^c^	21.73±3.07	22.59±3.82	0.28

a Values are given as mean±SD unless otherwise indicated

b Mann–Whitney U test

c Calculated as weight in kilograms divided by the square of height in meters

**Table 2:** Pain severity measured on a 10-cm visual analog scale

**Time**	**Before**	**1** ^st^ ** Cycle**	**2** ^nd^ ** Cycle**	**Level of** **Significance**
**Groups**	**Mean and SD**	**Mean and SD**	**Mean and SD**	
**Melissa**	7.63±1.70	2.79 ±1.73	2.00±1.43	**P<0.001** a
**Placebo**	7.09±2.04	3.49 ±1.62	3.03±1.61	
**P Value**	P=0.267^b^	P=0.016^b^	P=0.002^b^	

a Fridmantest& Wilcoxon test

b Mann Whitney test

**Table 3:** Extent of the reduction in pain severity as measured on a 10-cm visual analog scale

**Baseline to second cycle**	**Baseline to first cycle**	**Groups** a
5.61±1.89	4.92±2.19	**Melissa **
4.07±1.94	2.67±1.71	**Placebo **
0.001	0.001	**P value** b

a Values are given as mean±SD unless otherwise indicated

b Mann–Whitney U test.

## Discussion

Findings showed that Melissa capsules can reduce moderate to severe dysmenorrhea. Primary dysmenorrhea is caused by uterine contractions associated with ischemia. Women with dysmenorrhea suffer from increased uterine activity resulting in increased tone and frequency of contractions (20). Dysmenorrheal pain is spasmodic and colicky in nature like labor. Thus, using a compound spasmolytic property would be effective on pain(21).

No similar study with Melissa was found for this purpose;only one study has investigated the effect of *Salvia officinalis* and *Melissa officinalis*as infusion on duration and severity of menstrual pain(22). 

In another study, Akbarzadeh *et al.,* (2015) assessed the effect of MO capsule on the intensity of premenstrual syndrome in high-school girls. After 3 month of *intervention*, the intensity of physical, psychological and social symptoms significantly decreased in the MO group but no significant difference was found in the placebo group (23).

However, other researchers have used this herb in other conditions of muscle spasms, e.g. relaxant effects of lemon balm oil was studied on tracheal and ileal smooth muscles of the guinea pig and it was the most potent (17).In a similar study (2009), the relaxing effect as well as antispasmodic imposes of valerian extract on women's uterus undergoing hysterectomy were investigated in a laboratory setting. It was shown that valerian reduced the uterine cramps (24).

 Causing contractions of uterine smooth muscles during menstruation, prostaglandins may result in manifestations of the same nature in other parts including dyspnea due to bronchial narrowing, diarrhea because of increased peristalsis and hypertension due to vasoconstriction (20). In addition, antispasmodic properties of lemon balm in coronary arteries as well as bronchi were indicated (25). Therefore, in patients with asthma and digestive disorders, synthetic drugs such as NSAIDs can be replaced by this herb. 

In a clinical trial, Rouzbahani *et al., *compared the effects of thyme with mefenamic acid on primary dysmenorrhea. Thyme has the same antispasmodic effects as lemon balm by blocking calcium canal(26). Their results were in agreement with ours. Thus, it can be concluded that lemon balm is likely to have significant effects on decreasing dysmenorrhea severity. 

 Also, pain duration was assessed. Mean durations of pain had no significant difference before the intervention in the groups but, after 2 months of the intervention, a significant difference was found in this regard with shorter time of pain in the experimental group. Hence, lemon balm may also be effective on the duration of dysmenorrhea. Regarding the associated signs, no significant difference was found between the groups before the intervention in terms of their severity.

 Both placebo and lemon balm groups took less sedative after the intervention; however, the difference was statistically significant. The side effects of NSAIDs include digestive disorders such as nausea, dyspepsia and vomiting; renal toxicity as nephrotic syndrome; hyperkalemia; peptic ulcer; bleeding from the stomach as well as other parts and vertigo(27). In contrast, no complication was reported with regard to lemon balm consumption. Since lemon balm may be effective on systemic signs associated with menstruation, further related studies are suggested. 

This study revealed that lemon balm may have a significant influence on decreasing the severity of primary dysmenorrhea. With regards to this influence and lack of any side effect due to its consumption, it seems that lemon balm can be administered safely for the management of primary dysmenorrhea. 
